# Sociodemographic vulnerability to cold and stormy weather in relation to health and life

**DOI:** 10.1016/j.wace.2025.100840

**Published:** 2025-12

**Authors:** Y.T. Eunice Lo, Joanne L. Godwin, Ulrika Maude, Huw Day, Nicolas J. Timpson, Kate Northstone

**Affiliations:** a Cabot Institute for the Environment, University of Bristol, UK; b School of Geographical Sciences, University of Bristol, UK; c Elizabeth Blackwell Institute for Health Research, University of Bristol, UK; d Centre for Health, Humanities and Science, University of Bristol, UK; e Jean Golding Institute, University of Bristol, UK; f Bristol Medical School, University of Bristol, UK

**Keywords:** ALSPAC, Cold weather, Storms, Physical health, Mental health

## Abstract

Cold weather is the leading weather-related health threat in the UK, and winter storms can cause significant damage and disruption to society. However, their effects on the UK public’s daily life and health remain underexplored. Leveraging resources from the Avon Longitudinal Study of Parents and Children, we surveyed 2 736 individuals about their experiences and behaviour during a cold spell and Storms Henk, Isha, and Jocelyn in January 2024. Over half of respondents reported reduced physical activity during cold (51 %) and stormy weather (53 %), and worsened mood during the storms (57 %). Certain sociodemographic groups had more than twice the odds of reporting negative impacts compared to other groups during both cold and storms: females, individuals aged ≤34, and those with a disability reported worse appetite; unemployed and employed individuals (compared to retired respondents) reported lower productivity; and those with a long-term health condition or disability reported worse physical health and access to healthcare. While over 90 % of respondents were aware of severe weather warnings and more than 80 % took adaptive actions, enhanced public messaging on staying active indoors and seasonal vaccinations, and financial support for home heating are crucial to mitigate the adverse effects of cold and stormy weather.

## 1 Introduction

The UK winter health burden is known to be high, and it is compounded by surges from weather hazards such as cold spells and storms (Charlton-Perez et al., 2019; Curtis et al., 2017). On average, over 60, 000 deaths are attributable to cold temperatures in England and Wales each year (Gasparrini et al., 2022), and all-cause mortality risk increases by 9 % under extreme cold conditions in Scotland (Wan et al., 2022). While cold-related cardiovascular and respiratory diseases are by far the two largest sources of weather-related mortality in the UK; cold-related musculoskeletal health, and cold- and storm-related physical trauma and mental health issues also contribute to the winter health burden (Mitchell et al., 2024). There is limited expert consensus (i.e., more than one-third of 30 responses in an expert elicitation differing from the mode value) on the magnitude of the health burden arising from some of these lesser-considered weather-health issues (Mitchell et al., 2024).

Understanding the health impacts of cold and stormy weather is more complex than analysing mortality and morbidity data, as many impacts are pre-clinical and not available in routine National Health Service (NHS) records. Aspects of daily life such as physical activity and sleep quality may be affected by extreme weather directly and indirectly (e.g., through behavioural change during extreme weather), and they are closely linked to health and wellbeing outcomes, with potential economic implications (Botzen et al., 2019; Dell et al., 2014). Using alternative methods to collate individual experiences of adverse weather, behaviours, and contextual information of individuals that may alter their experiences, is important to fill these gaps and provide evidence for extreme weather alerting and adaptation guidance.

Large-scale surveys offer insights that can fill these evidence gaps. For example, data collected through the Longitudinal Survey of Australian Children showed that 12- and 13-year-olds (1100 young adolescents) engaged in less moderate- and vigorous-intensity physical activity and spent more time sedentary during cold or rainy weather, although their sleep remained largely unaffected (Nguyen et al., 2023). The American Time Use Surveys (42,280 respondents) suggested that individuals spent half an hour less on outdoor leisure activities at sub-zero temperatures, compared to around 25 °C (Zivin and Neidell, 2014). However, the equivalent UK literature, detailed in the next two paragraphs, lacks comparable large-scale data capturing relevant daily life and behavioural changes, particularly during cold weather.

A previous study interviewed 35 adults over the age of 65 in the Midlands and North of England within two days of a Level 3 cold weather alert in winter 2012/13 (Jones and Mays, 2016). They found that participants believed cold temperatures exacerbated pre-existing health conditions and were concerned about falling. Home owner-occupiers were more likely to report lower thermal comfort due to living in harder-to-heat homes compared to social housing tenants, and participants found advice and assistance from a charity more helpful than government assistance. Another study conducted semi-structured interviews with six care provider managers in Castlederg, which held the coldest temperature record in Northern Ireland from the winter of 2010 (Kennedy-Asser et al., 2025). Managers revealed that roads were impassable on days with heavy snowfall and frost, impacting staff’s ability to commute to work and to provide care for rural communities. A third study examined the consequences of a severe snowstorm in 1947 to a sheep farming community in Cwm Tywi, Wales (Jones et al., 2012). Interviews with people who experienced this cold event, revealed that community members stayed at home, stocked up food supplies, burnt wood to heat houses, and manually circulated water to prevent it from freezing. However, the severe snowstorm led to social isolation and loss of thousands of sheep, which eventually led to abandonment of the valley attributed to emotional distress.

Previous studies also employed surveys to understand the effects of storms on the general population. In one study, responses in the UK household longitudinal study (34,988 respondents) were linked to rainfall during the storms in winter 2011/12 (Berlemann et al., 2020). The authors concluded that extreme rainfall negatively affected respondents’ self-reported life satisfaction. In another study, self-reported psychological symptoms in an English National Study of Flooding and Health questionnaire three years after flooding in 2013/2014 were analysed (819 respondents). Significantly higher odds of probable depression and post-traumatic stress disorder were found in respondents who experienced flooding, compared to those unaffected (Mulchandani et al., 2020). In addition, according to the 2014–2015 Adult Psychiatric Morbidity Survey (7525 respondents), experiencing storm or flood damage to one’s home in the preceding six months was a risk factor of common mental disorder (Graham et al., 2019).

Building on the literature, and a recently developed extreme weather survey of the Avon Longitudinal Study of Parents and Children (ALSPAC) cohort that was piloted in the context of a 2023 heatwave (Godwin et al., 2025), we present a new study about UK residents’ lived experiences, vulnerability, and adaptative behaviours during cold and stormy weather in January 2024. This month was chosen as our case study because it was characterised by a significant cold and dry spell from the second week of the month, sandwiched between stormy and wet weather that occurred in the first and last weeks. In particular, the period between 14th and 20th January 2024 was anomalously cold with respect to the 1991–2020 average temperature for January, with the UK average temperature dipping to 2 °C below climatology (UK Met Office Press Office, 2024b). Amber Cold-Health Alerts (second highest warning level) were issued by the UK Health Security Agency (UKHSA) and Met Office for all regions of England, warning the public and the health sector of possible significant impacts on the whole health service for an extended period (UK Health Security Agency, 2024c). Yellow severe weather warnings for snow and ice were also issued by the Met Office for northern Scotland and Northern Ireland (UK Met Office Press Office, 2024a). Both Cold-Health Alerts (England only) and severe weather warnings (UK wide) follow a coloured, impact-based system. Yellow warnings (the lowest level) indicate either low likelihood or impact of the weather. Amber warnings (the middle level) indicate low, medium or high likelihood of high or medium impact, respectively. Red warnings (the highest level) indicate high likelihood and impact. The contextual meanings of the warnings issued for our studied cold spell and storms are summarised in Table 1.

Before and after this cold spell were three named storms: Storm Henk between 2^nd^ and 4^th^ January 2024, and Storms Isha and Jocelyn between 21^st^ and 24^th^ January 2024. Strom Henk brought damaging winds gusted well above 58 mph to southern England and Wales (peak gust = 94 mph on 2^nd^ January), and wet weather to nearly all parts of the UK (Kendon, 2024a). An Amber severe weather warning for wind was issued for southern England, and 233 flood warnings were in force across England and Wales (Morris, 2024). At least one person was killed, and another person was injured, and tens of thousands of properties experienced power cuts (Kendon, 2024a). Storm Isha brought strong winds gusted above 69 mph to the northern half of the UK (peak gust =124 mph on 21^st^ January) and very wet weather to western upland areas (Kendon, 2024b). Amber and Red severe weather warnings for wind were issued for half of the UK and northeast Scotland, respectively, along with 53 flood warnings in England and Scotland (Ambrose and Topping, 2024). At least two people died and hundreds of thousands of properties had power cuts (Kendon, 2024b). Storm Jocelyn was less severe for the UK in comparison (peak gust = 140 mph on 23^rd^ January, but only at a mountain summit), but its immediate arrival after Isha caused further disruption.

Against this meteorological backdrop, we examine how adverse winter conditions may have influenced aspects of daily life relevant to human health and wellbeing—including physical health, physical activity, sleep quality, appetite, mood, productivity, and access to healthcare. These metrics are not routinely captured in NHS datasets, allowing this study to contribute novel descriptive associations between adverse winter weather and daily life and behaviours in the UK. We investigate sociodemographic determinants of vulnerability to negative lived experiences and highlight the adaptive capacity of the UK public to severe winter weather. In doing so, we offer a valuable update to the national evidence base, much of which remains to be based on winter weather events from over a decade ago.

## 2 Methods

### 2.1 ALSPAC

Formerly known as Children of the 90s, ALSPAC is a world-leading cohort study that has been running since the early 1990s. Pregnant women resident in Avon, UK, with expected delivery dates between 1^st^ April 1991 and 31^st^ December 1992 were invited to take part in ALSPAC (Boyd et al., 2013; Fraser et al., 2013). Of the 20,248 eligible, 14,541 pregnancies enrolled. Eligible pregnancies that did not enrol at the beginning were invited to sign up when the children reached the age of 7 years, resulting in an increased sample size (Major-Smith et al., 2022; Northstone et al., 2019). 14,833 women, their 3807 enrolled partners (Northstone et al., 2023) and 14,901 children (alive at one year of age) have been followed ever since. This cohort now encompasses three generations: the original parents, the original children born in the 1990s (now in their mid-thirties), and the children of the children of the 90s. Extensive data have been collected from participants across multiple domains, including home and lifestyle factors; biological samples such as blood, urine, hair, nails, saliva, and placenta; genomic measures such as DNA sequence, gene expression, and methylation; as well as cognitive and behavioural data. These datasets are further enriched through linkage with external health, education, and environmental records. Please note that the study website contains details of all available data through a fully searchable data dictionary and variable search tool (ALSPAC, 2024).

### 2.2 Digital survey

We sent out an online survey in the form of Microsoft Forms to all ALSPAC participants with a valid email address on 5^th^ March 2024. The survey content was approved by the ALSPAC Ethics and Law Committee in February 2024, and it was adapted for the cold and stormy weather in January 2024 from a previous heatwave survey conducted by the same team (Godwin et al., 2025). Participants had two weeks to respond to this survey.

In the first section of the survey, we asked participants to think about the anomalously cold period between 14^th^ and 20^th^ January 2024 and rate their experience of eight aspects of life. These aspects were chosen as indicators of physical health (physical health, physical activity/exercise, appetite), mental health (sleep quality, mood), productivity (at home, at work) and access to GP, hospital or emergency care. In each of these aspects, participants could choose ‘a lot worse’, ‘slightly worse’, ‘no change’, ‘slightly better’, ‘a lot better’, or ‘n/a’ (i.e., not applicable), compared to normal. We then asked whether participants were aware of cold weather warnings (yes or no), and whether they made any changes to their usual daily activities in response to the warnings (yes or no), or in response to the cold weather itself (yes or no). Participants were given free text boxes to report any changes they made in response to advance warnings and to the weather separately, as well as a third free text box to tell us anything else about how they were affected by the cold period in question.

In the second section, we asked participants to think about Storms Henk, Isha, and Jocelyn that impacted the UK in January 2024 and answer the equivalent questions to the above, but for storms and flooding. In the third section, we asked for basic information about the participant, including their gender (female, male, non-binary), age group (25–34, 35–44, 45–54, 55–64, 65–74, 75–84, 85+), whether they had a disability (yes or no), and whether they had a long-term health condition (yes or no). We also asked about the type of housing (detached/semi-detached, terrace, flat, bungalow), residential area (urban, suburbs, rural), and UK region the participant lived in (Cymru Wales, East Midlands, East of England, London, North East & Cumbria, North West, Northern Ireland, Scotland, South East, South West, West Midlands, Yorkshire & the Humber). The last question asked about the participant’s employment status (employed, unemployed/not working, student, retired). For each question participants were also given the option of ‘prefer not to say’ and ‘other’ (free text). A limited number of characteristics were asked here to maintain participant confidentiality and enable a short, rapid survey methodology.

Finally, we asked the participant to confirm they understood that the information they provided in this survey would not be identified and attributed to them and, therefore, could not be deleted. The collected data would also not be linked to the rest of ALSPAC data. Full survey questions are included in Supplementary Information. Study data were collected and managed using REDCap electronic data capture tools hosted at the University of Bristol (Harris et al., 2009). REDCap (Research Electronic Data Capture) is a secure, web-based software platform designed to support data capture for research studies.

### 2.3 Statistical methods

After filtering out respondents who we deemed ineligible for this analysis, for example, those who lived abroad (5 individuals), we combined the responses ‘a lot worse’ and ‘slightly worse’ into a ‘worse’ category, in order to assess the influence of sociodemographic factors on people experiencing worse effects, compared to ‘no change’ (baseline). We also combined age groups into three categories to better reflect the overall ALSPAC cohort composition and to improve relevance for public health policy: ≤34 years (the original offspring), 35–64 years (original parents of the cohort; see Section 2.1) and ≥65 years (also parents but the vulnerable age group in UKHSA’s Adverse Weather and Health Plan (UK Health Security Agency, 2024a)).

For each aspect of life under each weather hazard (cold or storms), we constructed multinomial logistic regression models considering the participants’ responses as outcomes, with all sociodemographic factors (gender, age group, employment status, housing type, residential area type, disability, and long-term health condition) included as independent variables in the models to begin with. We then included an interaction term for gender and age. We chose the model with a lower Akaike information criterion (AIC) value as our starting model.

We then made stepwise deletions to remove one independent variable at a time, to eventually reach a minimum adequate model. This process involved choosing the model with the lowest AIC value at each step, and comparing this model with the previous one (that had one variable more) through an analysis of variance (likelihood ratio test). This process continued until the simplified model was different from the previous one at the 5 % significance level (at which point the previous model was chosen as the minimum adequate model). We used the variance inflation factor (VIF) to check for severe multicollinearity (VIF >10) and did not include any models with severe multicollinearity (Field et al., 2012). For all remaining independent variables in the minimal adequate model, we calculated their odds ratios from their regression coefficients. This analysis is based on the regression analysis published in (Godwin et al., 2025). As with the previous study, the analysis here is designed to be descriptive of broad associations between sociodemographic factors and survey responses, and it does not seek to determine causality (Major-Smith et al., 2023). This is because weather-related lived experiences are self-reported and may be subject to reporting bias, particularly if individuals with certain health or life conditions are more likely to perceive or recall changes during adverse weather (see Section 4). Additionally, this study’s cross-sectional data cannot control for unobserved, time-invariant individual characteristics that may be correlated with both exposure and outcomes. Odds ratios presented here indicate the change in the odds of answering ‘worse’ for a one-unit change in a sociodemographic factor (adjusting for other factors included in the relevant model).

### 2.4 Topic modelling on free text responses

We used natural language processing to extract common topics from free text survey responses about actions and changes made due to adverse weather and warnings. We did this for cold and storms separately. Specifically, we used BERTopic modelling, which was designed to sort a collection of documents of text into groups based on themes (Grootendorst, 2022) and had been used in a study related to extreme weather (Torricelli et al., 2023). BERTopic works on the basis that given a collection of numerical data, there are well-developed methods of assigning this data into different subgroups or clusters. Using pre-trained sentence transformers (we used the standard for BERTopic “all-Mini-LM-L6-v2”), one can convert text ‘documents’ into numerical representations, i.e. large dimensional vectors, where directions in the vector space are associated with certain characteristics.

We set up a standard BERTopic model and adjusted its parameters so that when loaded with the text responses from a previous heatwave survey (~3 000 responses; Godwin et al., 2025), the model outputs were consistent with that from the previous manual analysis. We then applied this model to our survey responses and fine-tuned the parameters further to obtain a list of extracted topics. Here, the longer a free text response is (and the more topics it mentions), the harder it is to accurately encode information about it in vector form. To account for this, we divided free text responses by common characters used to separate ideas such as commas, full stops, hyphens, semicolons and the word ‘and’. This means if a text response looked like: ‘*I used the heating more, stocked up on food and used a heated blanket’*, then we split this into the three inputs: ‘*I used the heating more’, ‘stocked up on food’, ‘used a heated blanket’*. We counted the number of unique respondents talking about each topic and not just the total number of times a topic was mentioned, by assigning the corresponding respondent index to each section of split text.

With the original text responses split into short phrases which typically mentioned one topic each and then represented in high dimensional vectors using sentence transformers, the next step was to sort these vectors into clusters. Due to the difficulty of high dimensional clustering, we followed standard practice and used Uniform Manifold Approximation and Projection (UMAP) to reduce the dimensions (McInnes et al., 2020) on our sentence vectors. We then applied density-based clustering, Hierarchical Density-Based Spatial Clustering of Applications with Noise (HDBSCAN) (McInnes and Healy, 2017), to sort our data of reduced dimensions into clusters. This method does not force every point into a cluster; it labels some points as ‘outliers’ when appropriate. The outcome of this step was that we had an understanding of which documents should be clustered together.

Next, we used class-based term frequency-inverse document frequency methods (Sammut and Webb, 2011), which are standard as part of BERTopic, to extract likely representations for each cluster. This works on the basis that a term is a good representation for a group of text responses if it appears frequently in that cluster but appears infrequently across in other clusters, relative to the average size of the clusters. During this process, we removed common stop words such as pronouns ‘I’ and ‘we’ using standard collections from Python packages such as NLTK (Bird et al., 2009). These stop words appeared frequently in text responses but were not representations of the adaptation actions taken. We also considered N-grams, which are likely combination of terms that provide more semantic meaning; for example, ‘stayed’ and ‘home’ combined into ‘stayed home’ as a potential topic representation. We followed the common practice of considering N =1, 2, 3, i.e. singular words, bigrams and trigrams. This means that our list of candidate representations for each cluster could be singular words or phrases of two or three words that commonly appear together.

There were proposed representation labels that were semantically nearly the same, for instance, the labels ‘used more heating’ and ‘using more heating’. Therefore, we used a Maximal marginal Relevance (MMR) model (Carbonell and Goldstein, 1998) to reduce the semantic similarity of representation labels. MMR takes the proposed representations and compares their similarity to one another. It then allows the user to control the diversity of the representations. Using MMR with a sufficiently tuned diversity threshold allowed us to collapse these representations into one.

Once we had an initial list of topics (i.e., clusters with representation labels), we categorised them into major and minor themes through discussion, informed by our research experience and judgement. In some cases, multiple topic clusters from our topic model were combined into a single theme. In this step, we also reduced the ‘outlier’ cluster size by manually reading through topics therein. We reassigned certain ‘outliers’ to relevant major or minor themes, where appropriate. Other outliers—such as phrases like ‘as above’, ‘etc.,’ and ‘e.g.’—were removed, as they did not represent adaptation actions related to extreme weather.

## 3 Results

### 3.1 Survey respondents

**Fig. 1:**
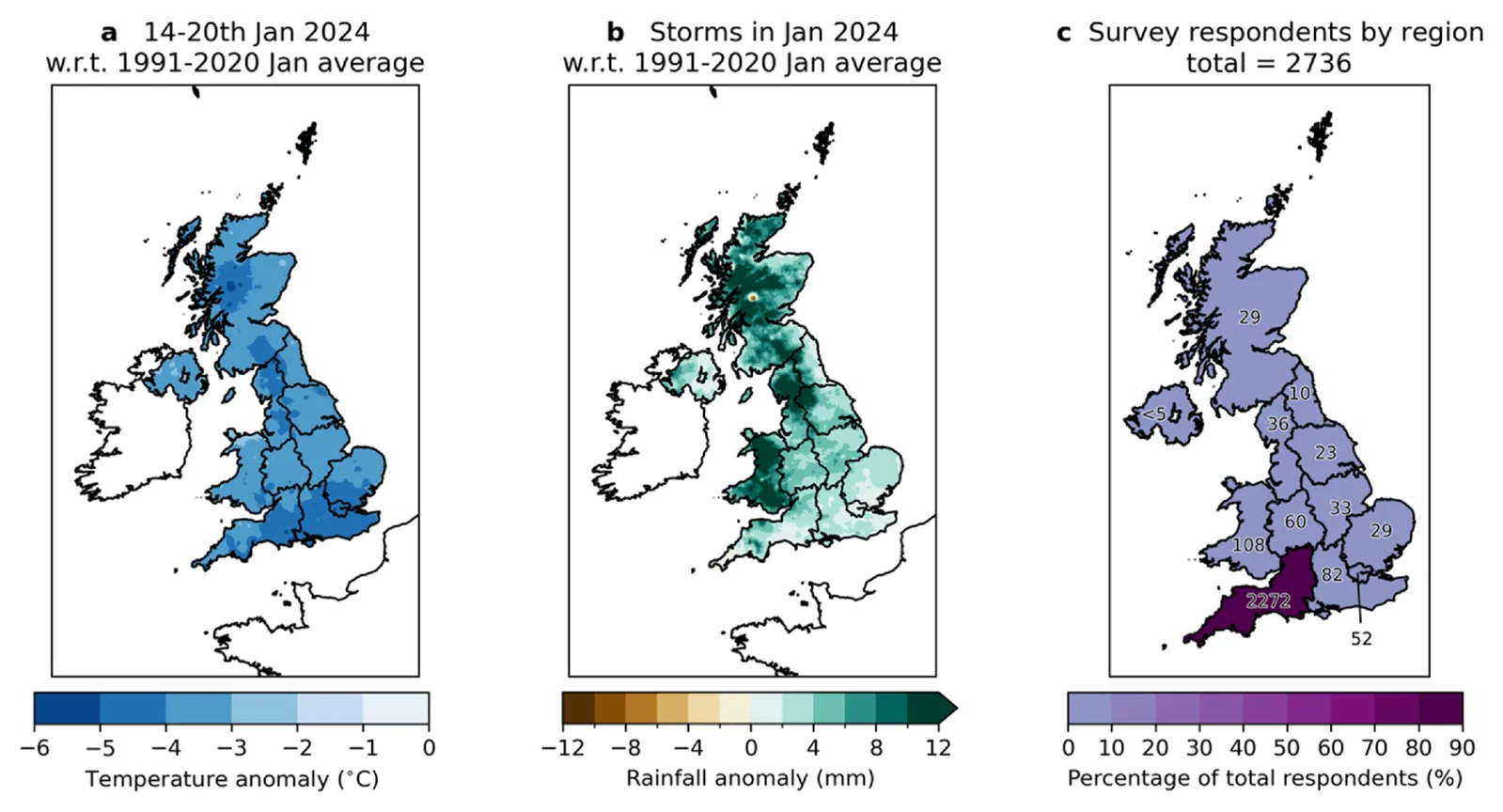
The studied extreme weather events and respondents’ locations in the UK. (a) Average temperature anomalies in the week of 14-20^th^ January 2024, with respect to the average January temperature in 1991–2020. (b) Average rainfall anomalies during Storm Henk (2^nd^ January 2024), Storm Isha (21–22^nd^ January 2024) and Storm Jocelyn (23–24^th^ January 2024), with respect to the average January rainfall in 1991–2020. (c) Number of survey respondents in each UK region (annotations), with colour shading representing the corresponding percentage of the total. The number for Northern Ireland is non-zero but fewer than five respondents; it is shown as “<5” to protect participant anonymity.

We received a total of 2736 valid responses from participants who were based in the UK during the extreme cold event and named storms (5 respondents did not live in the UK and were excluded from this number). Derived from daily average temperatures in the UK Met Office 1 km HadUK-Grid dataset (Hollis et al., 2019), Fig. 1a shows that the period 14^th^ to 20^th^ January 2024 was anomalously cold all over the UK, relative to the average January temperature in 1991–2020. In particular, South England, North West England and Scotland were up to 5 °C colder than climatology. Also based on 1 km HadUK-Grid data but for daily rainfall, Fig. 1b shows that nearly all parts of the UK were anomalously wet during Storm Henk, Storm Isha and Storm Jocelyn, compared to 1991–2020 climatology. Wales, North West England and Scotland had the largest rainfall anomalies.

Although some ALSPAC participants had moved out of the Bristol area since initial recruitment in the 1990s, Fig. 1c shows that 83 % of the respondents lived in South West England at the time of survey completion. The proportion of respondents living in other UK regions was far lower: 0.4 % in North East England, 1.3 % in North West England, 0.8 % in Yorkshire and the Humber, 1.2 % in East Midlands, 2.2 % in West Midlands, 1.1 % in East of England, 1.9 % in London, 3 % in South East England, 1.1 % in Scotland, 3.9 % in Wales, and <0.2 % in Northern Ireland. This means that even though our analysis had representation from all UK regions, most of the evidence of lived experience came from the South West, part of which experienced some of the largest cold anomalies in the country (Fig. 1a) but not large rainfall anomalies (Fig. 1b) in January 2024. We present results from all 2736 respondents in the main manuscript. We present results from a sensitivity analysis based on the subset of South West England respondents (83 %, i.e. 2272 individuals) in Supplementary Information, using the same Methods as Section 2.2.

**Fig. 2:**
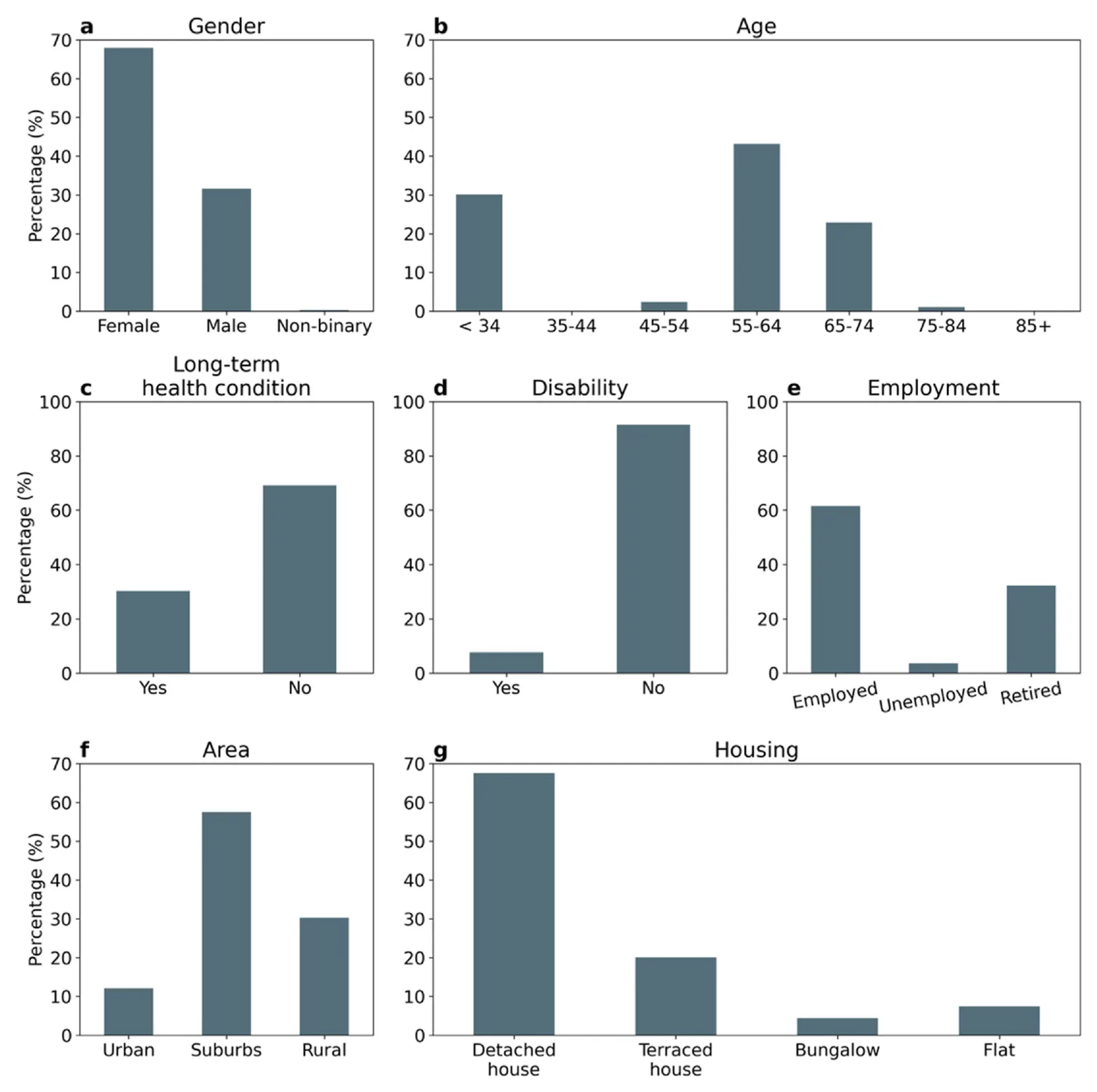
Summary of survey respondents’ characteristics. Percentage of 2 736 respondents in (a) gender, (b) age, (c) long-term health, (d) disability, (e) employment status, (f) residential area, and (g) housing categories.

Fig. 2 shows that 68 % of our survey respondents were female, with more female respondents in all age groups except the 75+ age group (data not shown). Less than 1 % of respondents identified as non-binary, or did not specify their gender. Survey respondents were predominantly in the 55–64 (43 %), 65–74 (23 %) and ≤34 (30 %) age groups, reflecting ALSPAC’s nature of being a parents and children (now in their 30s) study. About 30 % of respondents reported having a long-term health condition, and about 8 % reported having a disability. A majority (62 %) of respondents were employed or self-employed, 32 % were retired, and 4 % were unemployed or not working. More than half (57 %) of the respondents lived in suburban areas, and 30 % lived in rural areas. Only 12 % of respondents lived in urban areas in city or town centres. 68 % of respondents lived in a detached or semi-detached house, 20 % lived in a terraced house, 4 % lived in a bungalow, and 7 % lived in a flat.

### 3.2 Associations with health and life

**Fig. 3:**
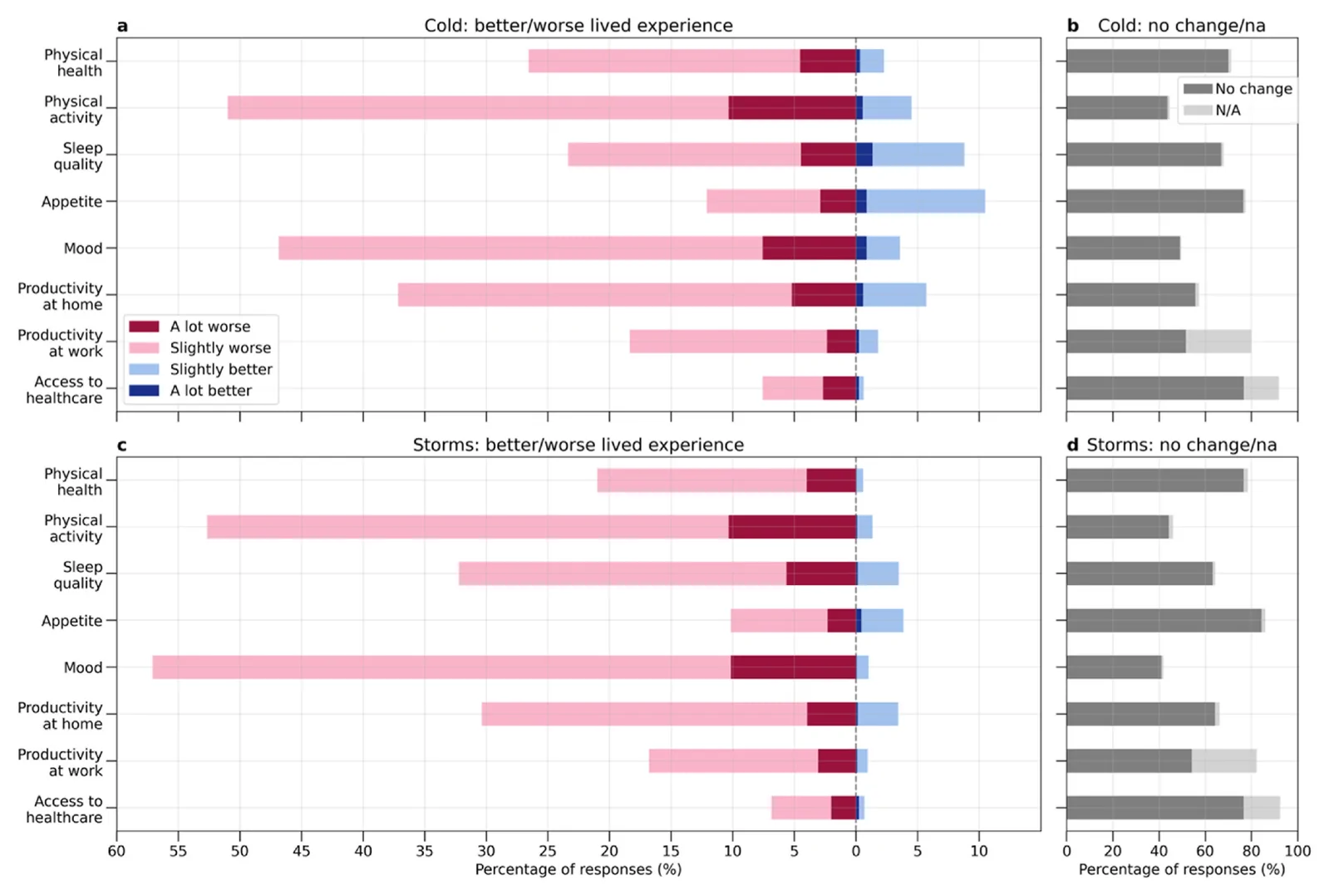
Lived experience of cold weather and storms in aspects of life. (a) Percentage of respondents reporting better (dark blue: a lot better; light blue: slightly better) or worse (dark red: a lot worse; light red: slightly worse) experience during cold weather. (b) Percentage of respondents reporting no change (dark grey) in aspects of life during cold weather, or aspects of life being not applicable (n/a; light grey) in the cold period. (c) Same as (a) but during three named storms. (d) Same as (b) but during three named storms.

Fig. 3a shows the proportions of total respondents who perceived better (light blue for ‘slightly better’ and dark blue for ‘a lot better’) and worse (pink for ‘slightly worse’ and dark red for ‘a lot worse) experiences during the cold period. In all aspects of life, more respondents reported an adverse experience from the anomalously cold weather than a positive experience. This was particularly evident for physical activity, for which over half (51 %) of all respondents reported worse experiences; mood, for which 47 % of respondents reported worse experiences; and productivity at home, for which 37 % of respondents reported worse experiences. In the other aspects of life, 27 % of respondents reported worse physical health, 23 % worse sleep quality, 18 % worse productivity at work, 12 % worse appetite, and 8 % experienced worse access to healthcare. For a very small proportion (1.5 %) of all respondents, the cold weather was associated with negative experiences across all aspects of their daily lives (not shown).

Optional free text responses that described physical and mental health changes offered an opportunity to understand these results. Among 185 relevant cold survey free text responses (not shown), ‘low mood’, ‘depressed’, ‘felt miserable’ and ‘low motivation’ were mentioned 47 times. Some of these were related to the weather itself, such as ‘felt so rubbish, no energy or motivation to do anything’, which could have had implications not only on the respondents’ mood, but also their physical activity and productivity. Some other comments were related to the impact of reduced physical activity on mood, such as ‘slightly depressed by … restricted golf and cycling’. We show that reduced activity was an adaptation action against cold weather in Section 3.4. Other free text responses described physical health changes. For instance, worsened arthritis and joint pain (24 mentions), worsened asthma and breathing (11 mentions), chilblains (7 mentions), Raynaud’s phenomenon (6 mentions), and dry skin and eczema (5 mentions). Some respondents’ health was affected in more than one way, with one person saying, ‘I have rheumatoid arthritis and bronchiectasis which are worse in the cold weather’.

While there were some positive reported experiences associated with cold, they came from 10 % to 9 % of all respondents with regard to appetite and sleep quality, respectively (Fig. 3a). In all other aspects of life, only ~5 % or less of all respondents reported positive experiences from cold weather. Only one respondent consistently reported positive experiences across all aspects (not shown). These statistics were reflected in the smaller number (total = 20) of optional free text responses that expressed a positive attitude towards the cold period. Some respondents enjoyed the cold, with one person saying, ‘I found it exhilarating’. Other respondents believed the cold to be beneficial to their health, including ‘I have very bad eczema, this improves with cold weather’ and ‘menopausal, so love the cold!’.

Some, often more than half, of respondents did not experience a change in an aspect of life due to cold weather (dark grey in Fig. 3b). This ‘no change’ proportion ranged from 44 % in physical activity to 77 % in access to healthcare, indicating resilience to cold weather potentially through adaptation. We discuss awareness to weather warnings and adaptation actions in Section 3.4. Overall, 15 % of all respondents consistently reported ‘no change’ to all aspects of life (not shown). Certain aspects of life, most notably productivity at work, did not apply to 28 % of respondents (light grey in Fig. 3b). This was roughly consistent with the fact that 32 % of our respondents were retired (Fig. 2e). About 15 % of respondents did not think access to healthcare was applicable to them during the cold period (Fig. 3b).

Fig. 3c and d shows the equivalent results for stormy weather, the reported adverse experiences of which outnumber positive experiences in individual aspects of daily life. Specifically, 57 % of respondents reported having slightly or a lot worse mood, and 53 % of respondents reported worse experiences in physical activity. In the other aspects of life, 32 % reported having worse sleep quality, 30 % reported having worse productivity at home, 21 % had having worse physical health, 17 % had having worse productivity at work. 10 % of respondents had worse appetite, and 7 % experienced worse access to healthcare. Only 1.8 % of all respondents consistently reported negative experiences across all life aspects of interest (not shown). This group largely consisted of respondents who also reported negative experiences across all aspects in cold weather.

We received 151 optional free text responses about the health changes associated with the January 2024 named storms (not shown). A vast majority of these were related to mood or mental health. First, 59 responses mentioned ‘low mood’ and feeling ‘depressed’, ‘fed up’ or ‘frustrated’. These were primarily related to reduced activity (also an adaptation action against storms, see Section 3.4). One person said they were ‘unable to exercise outside, leading to lower mood’, and another person said they had ‘more cabin fever’. Second, 47 responses described feeling ‘anxious’, ‘concerned’ or ‘frightened’. Most of these concerns were about damage to property such as ‘increased anxiety when very windy - concerned about roof tiles, fences, tree damage occurring’, whereas other concerns were about personal safety and other people. One respondent was ‘concerned for the public and farmers who would be impacted by the flooding’. The noise of high winds also led to poor sleep,according to 8 free text responses.

Positive self-reported experiences of the named storms in January 2024 were very few, reported by only 0.6 % (physical health) to 4 % (appetite) of all survey respondents (Fig. 3c). Across all aspects of life, two respondents reported positive experiences throughout (not shown). Of the 151 relevant free text responses about the storms, 11 were positive (not shown). One participant said, ‘storms improve my mood and I would usually be more likely to go running in them’, and another said, ‘I love the sound of heavy rain, I find it easier to fall asleep listening to it’. Together with the negative responses above, these comments demonstrate that weather affects different people differently, and sometimes in opposite ways.

As with cold weather, a proportion of respondents did not experience a change to daily life during the storms (Fig. 3d). We discuss respondents’ actions in response to the named storms and their warnings in Section 3.4. The ‘no change’ to daily life proportion ranged from 41 % in mood and 84 % in appetite. Considering all aspects of life together, 15 % of respondents reported ‘no change’ consistently (not shown). In addition, 28 % and 16 % of respondents did not think productivity at work and access to healthcare were applicable to them, respectively, during the storms. These ‘n/a’ proportions were nearly identical to those in the cold weather section, as the pool of respondents was the same.

### 3.3 Vulnerability to adverse experiences

#### 3.3.1 Cold

**Fig. 4:**
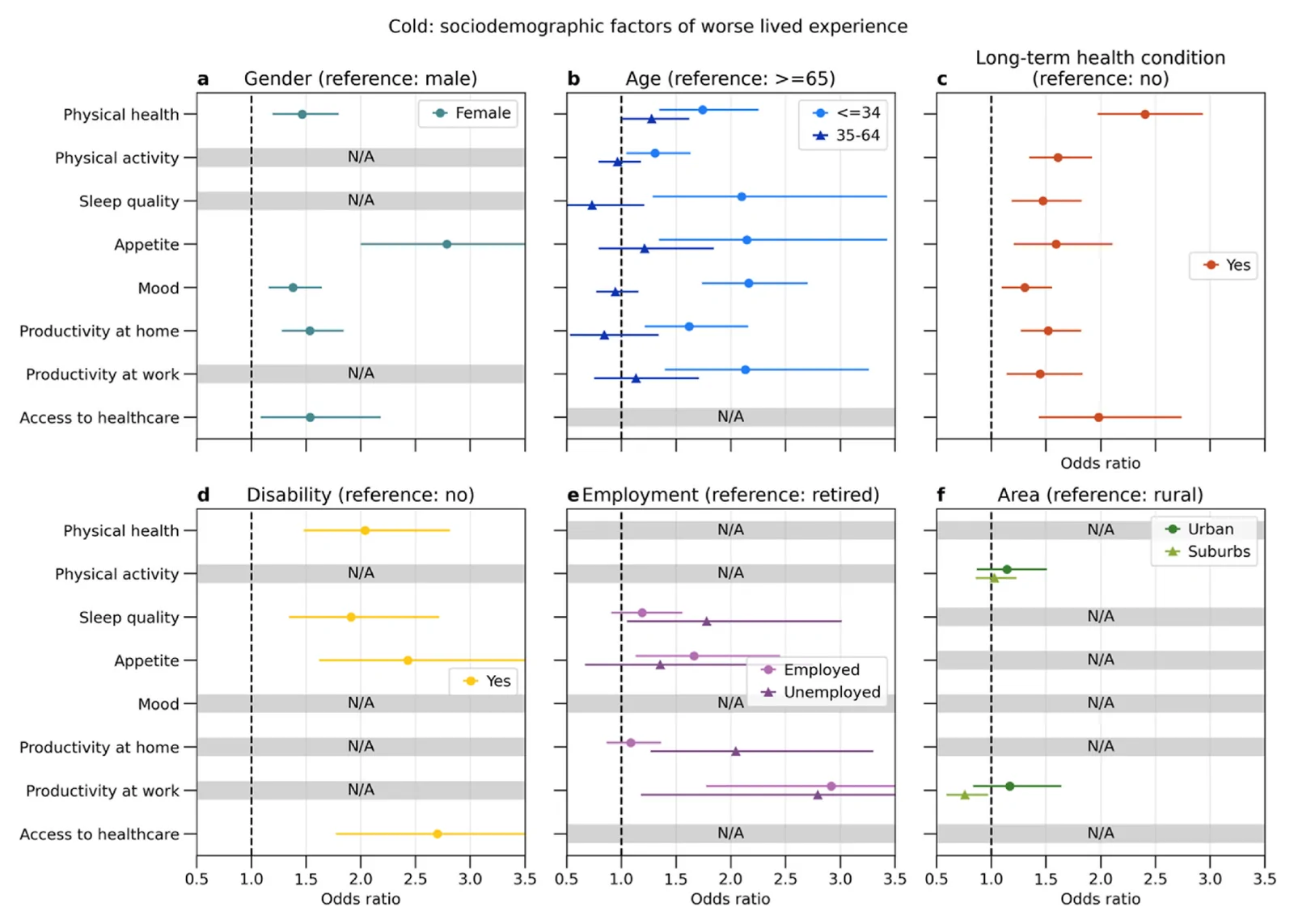
Odds ratios of reporting worse lived experience during cold weather in sociodemographic groups compared to a reference group. The error bars indicate the 95 % confidence intervals. (a) Female compared to male. (b) The ≤34 (light blue circle) and 35–64 (dark blue triangle) age groups, compared to the over 65s. (c) Respondents with a long-term health condition compared to those without. (d) Respondents with a disability compared to no disability. (e) Employed (light purple circle) and unemployed (dark purple triangle) respondents, compared to retired respondents. (f) Respondents living in urban (dark green circle) and sub-urban (light green triangle) areas, compared to rural areas. N/A indicates where a sociodemographic factor is not included in the final regression model.

**Fig. 5:**
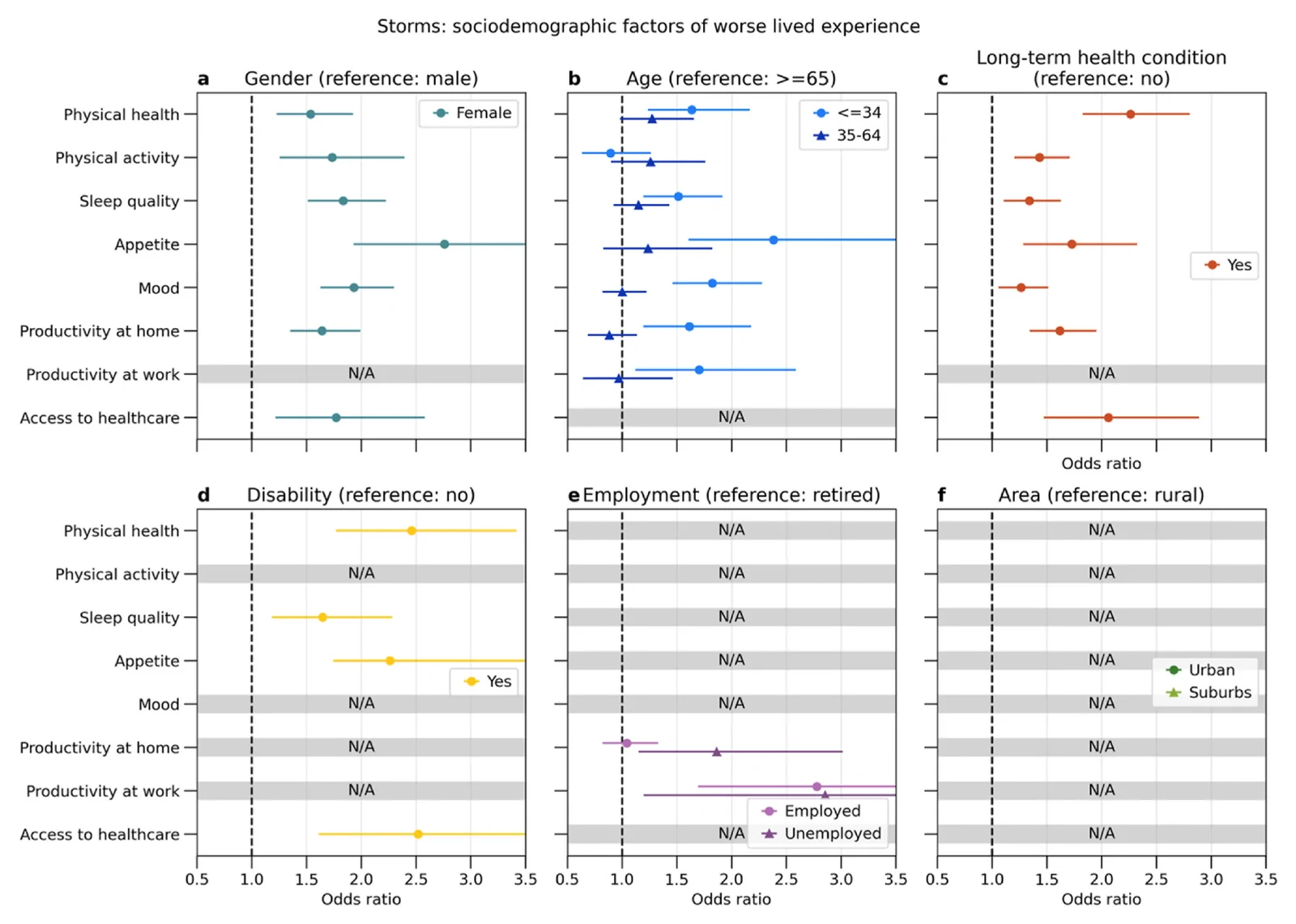
Odds ratios of reporting worse lived experience during the storms in sociodemographic sub-groups compared to a reference. (a) Female compared to male. (b) The ≤34 (light blue circle) and 35–64 (dark blue triangle) age groups, compared to the over 65s. (c) Respondents with a long-term health condition compared to those without. (d) Respondents with a disability compared to no disability. (e) Employed (light purple circle) and unemployed (dark purple triangle) respondents, compared to retired respondents. (f) Respondents living in urban (dark green circle) and sub-urban (light green triangle) areas, compared to rural areas. N/A indicates where a sociodemographic factor is not included in the final regression model.

Given that worse lived experiences outnumber improved experiences in all studied aspects of life, we report which sociodemographic groups were associated with adverse experiences more often than other groups. Fig. 4 shows the odds ratios (ORs) and 95 % confidence intervals (CIs) of reporting worse lived experience (‘slightly worse’ and ‘a lot worse’ combined, versus ‘no change’) for each aspect of life during cold weather for respondents in sociodemographic groups, compared to the corresponding reference group. The sociodemographic characteristics associated with differences in reported experiences, and therefore are included here, are gender, age, long-term health condition, disability, employment status, and residential area. Housing type (Fig. 2g) is not shown because it is not an independent variable in any of the minimum adequate regression models (see Table S1 in Supplementary Information). Where a sociodemographic characteristic is not an independent variable of the responses about an aspect of life, it is shown as ‘N/A’ in Fig. 4 (e.g., gender is not a variable of the responses about physical activity). The same applies to Fig. 5 for stormy weather in Section 3.3.2.

Gender had an influence on reported experiences on five of the eight aspects of life during anomalously cold weather (Fig. 4a): physical health, appetite, mood, productivity at home, and access to healthcare. Female respondents had greater odds of reporting worse lived experiences than male respondents in all these aspects of daily life, with an odds ratio of ~1.5 for physical health (CI = 1.2 to 1.8), mood (OR = 1.4, CI = 1.2 to 1.6), productivity at home (CI = 1.3 to 1.8), and access to healthcare (CI = 1.1 to 2.2). Moreover, female respondents had 2.8 (CI = 2 to 3.9) times greater odds of reporting worse appetite than male respondents. These were all significant at the 5 % level (all *p* values are listed in Table S2 in Supplementary Information). Due to the small number of non-binary respondents (Fig. 2a), we are unable to provide odd ratios for this group.

Age also played an important role in perceived changes on all aspects of life except access to healthcare (Fig. 4b–Table S2). Respondents who were 34 years old or younger were more likely to report worse lived experiences during cold weather, than respondents who were over 65 years old. Most notably, those who were 34 years old or younger had 2.1 (CI = 1.3 to 3.4) times greater odds of reporting worse sleep quality and appetite, 2.2 (CI = 1.7 to 2.7) times greater odds of reporting worse mood, and 2.1 (CI = 1.4 to 3.3) times greater odds of reporting worse productivity at work, than those who were 65 or above. Results comparing the 35–64 age group with the over 65 group generally suggest no differences, except for physical health (OR = 1.3, CI = 1 to 1.6). Combined gender and age (i.e., these variables’ interaction) was only a variable of sleep quality in the minimal regression models (Table S1), with results showing that females in the 35–64 age group tended to have worse sleep quality (OR = 2.5, CI = 1.7 to 3.6, compared to males in the over 65 age group; Table S2, not shown in Fig. 4).

Having a long-term health condition increased the odds of reporting worse experiences in all aspects of life during cold weather (Fig. 4c). The odds ratios were ~1.5 for physical activity (OR = 1.6, CI = 1.3 to 1.9), sleep quality (CI = 1.2 to 1.8), appetite (OR = 1.6, CI = 1.2 to 2.1), mood (OR = 1.3, CI = 1.1 to 1.6), productivity at home (CI = 1.3 to 1.8) and at work (CI = 1.1 to 1.8). Vulnerability to adverse cold-related changes in physical health and access to healthcare was even higher, with the respective odds ratios being 2.4 (CI = 2 to 2.9) and 2 (CI = 1.4 to 2.7) for respondents who had a long-term health condition, compared to those who did not. Those who had a disability had 2 times greater odds of having worse physical health (CI = 1.5 to 2.8) and sleep quality (CI = 1.3 to 2.7), 2.4 (CI = 1.6 to 3.6) times greater odds of having worse appetite, and 2.7 (CI = 1.8 to 4.1) times greater odds of experiencing worse access to healthcare, compared to respondents who did not have a disability (Fig. 4d).

Employment is an indicator of socioeconomic status, and our results suggest that it contributed to vulnerability to worse sleep quality, appetite, productivity at home and at work during cold weather (Fig. 4e). Respondents who were unemployed or not working had ~2 times greater odds of reporting worse sleep quality (OR = 1.8, CI = 1.1 to 3) and productivity at home (CI = 1.3 to 3.3). They also had 2.8 (CI = 1.2 to 6.6) times greater odds of experiencing worse productivity at work, than respondents who were retired. Although this group of respondents were either unemployed or not in work, ‘work’ here may include unpaid work such as caring for the elderly or children and volunteering work. On the other hand, being employed or self-employed, versus being retired, did not appear to influence adverse changes in sleep quality and productivity at home. However, respondents who were employed or self-employed had respectively 1.7 (CI = 1.1 to 2.5) and 2.9 (CI = 1.8 to 4.8) times greater odds of having worse appetite and productivity at work, compared to retired respondents.

Living in urban areas (e.g., city and town centres) versus rural areas (e.g., rural, countryside and coastal areas) did not appear to influence the odds of adverse lived experiences during cold weather (Fig. 4f). Respondents in suburban areas tended to be less likely to report reduced productivity at work than those who lived in rural areas, with an odds ratio less than one (OR = 0.8, CI = 0.6 to 0.97).

#### 3.3.2 Storms

Sociodemographic vulnerability to adverse experiences from the January named storms was similar to that from cold, as shown in Fig. 5. Gender, age, long-term health condition, disability and employment status were all factors influencing reported changes in aspects of daily life during the stormy weather (see Table S3 in Supplementary Information). For gender, female respondents had 1.5 to 2 times greater odds of reporting worse physical health, physical activity, sleep quality, mood, productivity at home, and access to healthcare than male respondents (Fig. 5a). The *p* values of these results are all below the 5 % level (see Table S4 for full model outputs). Again, similar to the cold results, females were 2.8 (CI = 1.9 to 3.9) times more likely to have experienced worse appetite than males during stormy weather.

Respondents who were ≤34 years old were 1.5 to 2 times more likely to report worse physical health, sleep quality, mood, productivity at home, and productivity at work in stormy weather, compared to respondents who were over 65 years old (Fig. 5b). Moreover, this youngest age group had 2.4 (CI = 1.6 to 3.5) times greater odds of reporting worse appetite than the oldest age group during stormy weather. However, there was no detectable difference in the odds of reporting worse physical activity between the youngest (≤34) and oldest (≥65) age groups. Comparisons of the 35–64 age group with the over 65 age group did not suggest detectable differences in any of the life aspects. Combined gender and age (i.e., these variables’ interaction) was only a variable of physical activity in the minimal regression models (Table S3), with results showing that females in the 35–64 age group tended to have lower odds of reporting worse experience (OR = 0.6, CI = 0.4 to 0.9 compared to males in the over 65 age group; Table S4, not shown in Fig. 5).

Those who had a long-term health condition were 1.3 to 1.7 times more likely to report worse experiences in physical activity, sleep quality, appetite, mood, productivity at home, than those who did not have a condition (Fig. 5c). Moreover, this vulnerable group had 2.3 (CI = 1.8 to 2.8) and 2.1 (CI = 1.5 to 2.9) times greater odds of reporting worse physical health and access to health care, respectively, during the named storms, than those who did not have a long-term health condition. Respondents who had a disability also had over 2 times greater odds of reporting worse physical health (OR = 2.5, CI = 1.8 to 3.4), worse appetite (OR = 2.3, CI = 1.7 to 3.9), and worse access to healthcare (OR = 2.5, CI = 1.6 to 3.9), than respondents who did not have a disability. The odds ratio for sleep quality was lower (Fig. 5d).

Respondents who were unemployed or not working had 1.9 (CI = 1.2 to 3) and 2.9 (CI = 1.2 to 6.8) times greater odds of reporting worse productivity at home and at work, respectively, than respondents who were retired (Fig. 5e). Perhaps unsurprisingly, respondents who were employed or self-employed had 2.8 (CI = 1.7 to 4.6) times greater odds of reporting worse productivity at work, compared to retired respondents. There was no detectable difference in the odds of reporting worse productivity at home between the employed and retired groups. Unlike cold weather, residential area (urban or suburban, versus rural) was not an independent variable of survey responses in any of the life aspects with regard to stormy weather (Fig. 5f and Table S3).

### 3.4 Warning awareness and adaptation

**Fig. 6:**
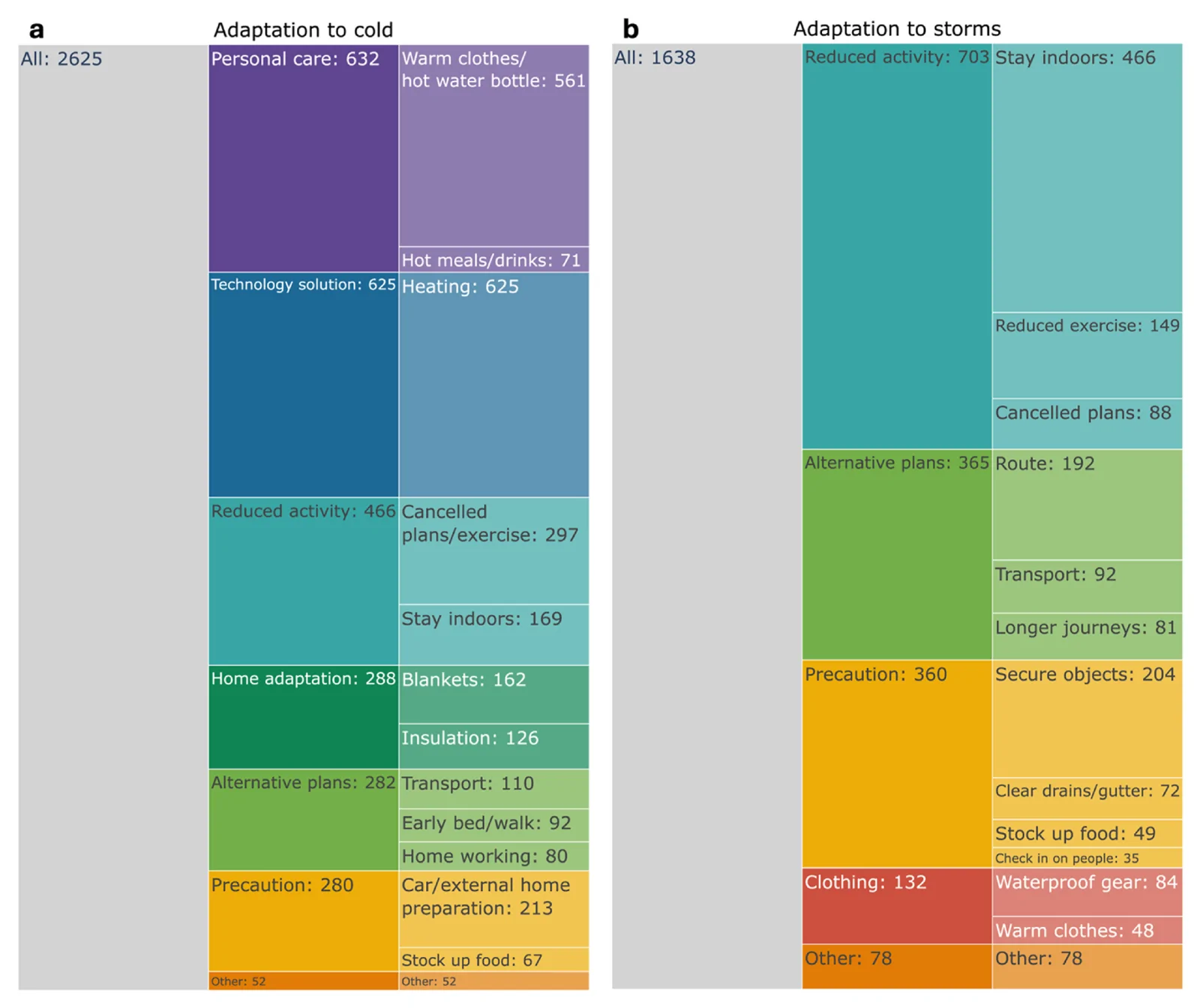
Adaptation actions by survey respondents. Adaptation actions in response to (a) cold weather and warnings and (b) stormy weather and warnings, clustered into major themes (middle column) and minor themes (right column). In both panels, the number in each theme indicates the total number of unique mentions of actions within the theme (e.g., repeated mentions of an action in response to the same weather hazard by the same respondent only count as one action). The vertical length of each theme is proportional to the number of actions within it, so themes with few actions may be difficult to read. Examples include “Other: 52” in the orange box at the bottom of panel (a), and “Check in on people: 35” in the yellow box within “Precaution: 360” in panel (b).

Early warnings and taking actions before and during extreme weather can reduce its impacts on daily life. In this section, we investigate the level of early warning awareness and adaptation in this cohort. 90 % of our respondents said they were aware of the cold weather warnings in January 2024 (Table 1), and 81 % said they made changes in response to the warnings. A smaller proportion, 62 %, of respondents made changes in response to the cold weather itself. We unpack the types of actions taken by these respondents in Fig. 6a, by counting the number of times actions were mentioned in response to either the warnings or the weather itself. All unique actions by each respondent who provided an answer were counted (some respondents took more than one action).

Adaptation actions for cold weather fell into six major themes. The first and largest theme was personal care by, for instance, wearing extra layers, thermal clothing, gloves and hats, and using a hot water bottle (n = 561). Personal care also included consuming warm meals such as soup, porridge, casseroles and hot drinks (n = 71). The second theme was putting central heating up or for longer, or using log burners to keep warm (n = 625). The third theme was reduced activity, which included cancelling appointments and doing less exercise (n = 297), and staying indoors (n = 169). The fourth theme was home adaptation by using extra blankets, duvets and bedding (n = 162), and insulating the home by closing curtains, windows and using draught excluders (n = 126). The fifth theme was alternative plans, for example, changing the mode of transport (n = 110), going to bed or for a walk earlier than usual (n = 92), and working from home (n = 80). Finally, some respondents took precautions such as defrosting the car and lagging pipes (n = 213) and stocking up on food supplies (n = 67).

Regarding Storms Henk, Isha and Joycelyn, 93 % of respondents were aware of warnings, and 85 % made changes because of the warnings. 74 % of respondents made changes in response to the stormy weather itself. Fig. 6b shows that adaptation actions for the storms fell into four major themes: reduced activity, alternative plans, precaution and clothing. Reduced activity was by far the largest theme, within which actions include staying indoors or at home (n = 466), doing less exercise outside (n = 149), and cancelling appointments and plans (n = 88). Alternative plans included taking a different route for commuting (n = 192), driving or taking the bus instead of cycling or walking (n = 92), and allowing extra time for journeys (n = 81). Precaution included securing garden furniture, bins and other outside items (n = 204), clearing drains and gutters (n = 72), stocking up on food (n = 49), and checking in on the elderly (n = 35). Finally, 84 respondents wore waterproof gear, and 48 respondents wore extra layers of clothing.

## 4 Discussion

We have shown that adverse experiences in health and life outnumbered positive experiences during the cold and stormy weather in January 2024 (Section 3.2). We have also shown that within the ALSPAC cohort surveyed, female respondents, the youngest (≤34) age group, people who had a long-term condition or a disability, and those who were unemployed or not working, were more likely to be vulnerable to these adverse experiences than other groups (Section 3.3).

Our results are broadly consistent with the vulnerable groups in UKHSA’s Adverse Weather and Health Plan (AWHP) and UK devolved nations’ weather-health plans, where the old and the very young, people who have pre-existing medical conditions and other health or economic circumstances that put them at greater risk of adverse weather harm are highlighted as vulnerable (Public Health Scotland, 2024; UK Health Security Agency, 2024a). Our results differ from the vulnerable groups in these plans in two ways. First, we have demonstrated that female respondents were at least 1.5 to 2.8 times more likely to report experiencing adverse cold and storm effects on physical health, appetite, mood, productivity and access to healthcare, as well as storm effects on physical activity and sleep quality, than male respondents. There is also some evidence in the literature that female adults are generally more sensitive to cold conditions than males (Jowkar et al., 2020). Yet only pregnant women are highlighted as vulnerable to cold weather in public health plans due to its association with late pregnancy, pre-eclampsia and preterm birth (Health and Social Care Public Health Agency for Northern Ireland, 2024; Hill et al., 2024; The Scottish Government, 2017; UK Health Security Agency, 2024b). Our results on gender, therefore, provide evidence to public health agencies that females, not only pregnant women, are likely to be more vulnerable to adverse cold and stormy weather impacts on health and daily life than males.

Second, our results on age vulnerability, i.e., the ≤34 age group being generally more vulnerable than the above 65 group, differ from these plans and previous papers that showed higher cold-related morbidity (Rizmie et al., 2022) and mortality (Gasparrini et al., 2022; Mitchell et al., 2024; Wan et al., 2022) risks in the above 65 age group. This demonstrates a dissonance between self-reported health and life experiences, the focus of this paper, and hospital and mortality statistics. Older adults tend to have lower thermal sensation and be less responsive to temperature changes than younger adults, due to a combination of physiological and lifestyle factors (van Hoof et al., 2017). Indeed, some of our respondents mentioned retirement as a reason why they were not impacted by the adverse weather, with one person saying, ‘I confess I quite like a cold snap now I am retired!’. Previous research also found that older adults tended to perceive their risks of experiencing illness from cold (and hot) weather as low (Ratwatte et al., 2022; Turner et al., 2024). Reasons for this included not self-identifying as vulnerable, ‘old’ or ‘frail’; believing past weather events were more severe because they felt subjectively worse or had fewer adaptation resources in the past; and having the ‘just put up with it’ attitude. These physiological, lifestyle and perception differences in older adults may explain why our results on age vulnerability differ from the vulnerability seen in health records. We discuss the limitations of our survey-based study in the latter part of this section.

In the free text responses, 24 people mentioned worsened arthritis and joint pain as adverse physical health outcomes of cold weather (Section 3.2). This may be explained by low temperatures causing muscles to contract and stiffen, ligaments to lose elasticity, blood vessels to narrow, and synovial fluid activity to slow down, all of which can contribute to joint pain and inflammation (Spear, 2025). However, research using citizen science on a smartphone application (2658 patients) found higher odds of pain in higher relative humidity, higher wind speed, and lower atmospheric pressure (Dixon et al., 2019). They did not find a relationship between pain and temperature per se, but they highlighted substantial individual differences, which were what our free text survey sections were designed to explore.

England and Northern Ireland’s public health agencies advise the public to heat rooms to at least 18 °C during cold weather, and the Welsh and Scottish Governments have higher indoor temperature recommendations (18–23 °C) for vulnerable households (Clair and Baker, 2022). These guidelines generally recommend people to also reduce draughts, close windows, wear several layers of clothing, keep in touch with vulnerable people, stock up on food and medication, get vaccinated for flu and COVID-19, and keep active indoors (Health and Social Care Public Health Agency for Northern Ireland, 2024; UK Health Security Agency, 2024d). We have shown that survey respondents took most of these actions for cold weather and some of the actions for stormy weather too (Section 3.4). They took additional measures such as reducing activity and changing plans. While survey responses about reducing activity were primarily about reducing outdoor exercise, very few mentioned keeping active indoors. There was also no mention of vaccination in survey responses. This suggests that public messaging about moving and stretching indoors to keep warm and reduce the risk of developing medical problems, as well as vaccinating against respiratory illnesses to reduce winter health burden, may need to be strengthened.

Whilst keeping homes warm is the main advice for staying healthy in cold weather across all UK nations, topic modelling of the free text responses of our cold survey has identified the cost of heating as a barrier to adaptation. 73 respondents mentioned financial constraints or worries about heating bills, and one person even said, ‘inside home temperatures dropped to 6 °C’ (not shown in Section 3.4). Other respondents said they went to the office to use the heating there. These respondents account for about 3 % of our study respondents, but the ALSPAC cohort is known to have higher household income, home ownership rate, and education attainment than the UK average (Boyd et al., 2013; Fraser et al., 2013). These suggest generally higher socio-economic status (and lower related vulnerability) in our respondents than the wider population, although a previous study found that home owner-occupiers had lower thermal comfort in cold weather than social housing tenants (Jones and Mays, 2016). This was because homeowners tended to live in older and harder to heat homes (total interviewees = 35).

Fuel poverty is an issue in the UK. A previous survey on poverty in Wales found that 31 % of sampled adults (total = 1 029) went without heating in their homes in the three months leading up to January 2024 (The Bevan Foundation, 2024). The most recent UK fuel poverty rates ranged from 13 % of households in England to 31 % in Scotland (Hinson and Bolton, 2024). Interventions like ‘Warm Home Prescription’, through which low-income patients with chronic conditions are offered financial support for heating, have shown to be useful in keeping these patients’ homes at recommended temperatures, increasing self-reported health and life satisfaction, and lowering their use of primary care services (Smith and Wilson, 2023). For every £1 spent on this scheme, £5.1 of wellbeing social value to patients was achieved in a trial that supported 823 patients in England and Scotland (Smith and Wilson, 2023). Scaling up schemes like this, as well as supporting long-term home energy improvements (Seaton-O’Connor et al., 2024), will help a financially vulnerable group overcome barriers to cold adaptation.

The UK Met Office advise the public to secure loose objects, fasten windows and doors, check roof tiles and fences, and clear guttering before a storm (UK Met Office, 2024b). They further advise people to stay indoors, avoid driving, and slow down and take care if driving is necessary, during a storm. Responses to our survey about the January 2024 named storms indicate that nearly all these actions were taken by a proportion of respondents (Section 3.4). Additionally, some respondents stocked up on food supplies, checked in on vulnerable people, and wore waterproof and warm clothing. These results suggest that public messaging combined with common sense may have made the UK generally adapted to stormy weather. Nevertheless, more severe storms have occurred since January 2024, most notably Storm Darragh in December 2024, for which the Met Office issued a rare Red warning for wind in Wales and South West England. Three million people in these areas received a siren-sounding emergency alert from the Government on their mobile phones. Future research is needed to understand the health and life impacts of this storm, the impact of the red warning on reducing incidents and protecting lives, as well as public opinion on emergency mobile phone alerts.

This study has made use of the generous involvement of ALSPAC participants as well as pre-existing data collection infrastructure in ALSPAC to gather 2 736 responses about the cold and stormy weather in January 2024. As far as we know, this is the largest survey of its kind in the UK. The large number of responses has allowed us to analyse them both quantitatively and qualitatively, generating real-life insights that are not captured in hospital or mortality data but are useful to inform extreme weather public health messaging and adaptation policies. We have also utilised one of the newest topic models based on natural language processing, BERTopic, to put free text responses about adaptation actions into themes (Sections 2.3 and 3.4). This is a major advancement in automating large-scale analyses of text responses in weather surveys, for previous UK studies either analysed far fewer qualitative answers (Jones and Mays, 2016), or used substantial human resources to conduct thematic analyses manually (Godwin et al., 2025). By testing our model on the responses from a previous heatwave survey and then fine-tuning its parameters for this study, we have set up a robust model that can be adapted by researchers for future use. The code and instructions are publicly availability (see Data availability).

However, this study has limitations. First, 83 % of our respondents were in South West England at the time of adverse weather (Section 3.1). Although we had representation from all regions in the UK, our results were skewed towards the South West. We conducted a sensitivity analysis based only on the South West England data subset, and our main results did not change (Supplementary Fig. S1–3). Future work should endeavour to include more participants from other UK regions, potentially through collaborating with other health cohorts.

Second, this study has relied on self-reported experiences in health and life, which are subjective and can be influenced by factors such as age differences in risk perception (see above). These reported experiences have also been designed as changes relative to the respondents’ ‘normal’ life (see survey questions in Supplementary Information), which is bound to vary with their circumstances. Future research could deploy wearables such as smart watches to track participants’ physical activity, body temperature, sleep patterns, and emotions, among other metrics, before and during an extreme weather event (Nguyen et al., 2023). This could be done in conjunction with asking participants about their qualitative experiences via a similar survey. By doing so, researchers will be able to gain a full picture of objective and subjective weather impacts on people’s health and life.

Third, we focused on the cold weather and storms of January 2024 because this period was uniquely affected by both weather hazards of interest, and because Storm Isha was the only storm that winter (December 2023 to February 2024) that had a Red warning for wind. However, the January cold spell followed an earlier episode in early December (UK Met Office, 2024a), suggesting that participants may have already been better prepared for cold conditions than at the start of the season. Furthermore, asking about three successive storms in the same survey limited our ability to explore how participants’ perception, lived experiences, and adaptation actions evolved over the course of the winter. Future studies will benefit from examining individual events occurring at the start through to the end of a season. By conducting the first winter weather survey within ALSPAC, we have established a precedent and provided a methodological foundation that will support a wide range of future research on public responses and adaptation to adverse weather.

## 5 Conclusion

By conducting a survey about the cold UK weather and named storms in January 2024 with ALSPAC participants, we conclude that the adverse weather was associated with more negative than positive changes in respondents’ health and life. More than half of our respondents (total = 2 736) had worse physical activity during both cold and stormy weather, and worse mood during the storms. We highlight sociodemographic vulnerability in groups whose odds of reporting negative experiences were at least 2 times that of other groups. For example, females having worse appetite in both cold and storms; ≤34-year-olds having worse sleep quality, productivity at work and mood in cold weather, and appetite during both cold and storms; and unemployed respondents having worse productivity and employed respondents having worse productivity at work, than retired respondents during both cold and storms. Long-term health conditions and disability made respondents more vulnerable to adverse cold- and storm-related changes in physical health and access to healthcare. Moreover, respondents with a disability reported having worse sleep quality during cold weather, and worse appetite during both cold and storms. While most respondents took adaptation actions, public health recommendations on keeping active indoors and vaccination were not reflected in survey responses. Increased public messaging and financial support for those in need to heat their homes, are important to reduce the impacts of cold and stormy weather on health and daily life.

## Supplementary Material

**Appendix A.Supplementary data**

Supplementary data to this article can be found online at https://doi.org/10.1016/j.wace.2025.100840.

Appendix A. Supplementary data

## Figures and Tables

**Table 1:** The relevant weather warnings issued for the studied cold spell and storms, and their contextual meanings. Severe weather warnings for snow, ice and wind, as well as Cold-Health Alerts, follow a coloured impact-based system. Flood warnings do not follow this system.

Hazard	Warning		Contextual meaning
Cold		Yellow warning for Snow	Low likelihood of medium impacts, e.g., travel disruption, rural communities being cut off, and power cuts.
		Yellow warning for Ice	Medium likelihood of low impacts, e.g., injuries from slips and falls, and icy patches on roads.
		Amber Cold-Health Alert	Impacts are likely to be felt across the whole health service, with potential for the whole population to be at risk.
Storms		Amber warning for Wind	Medium likelihood of medium impacts, e.g., building damage, road closures, power cuts, injuries and danger to life.
		Red warning for Wind	High likelihood of high impacts, e.g., building damage, road closures, power cuts, flying/washed up debris, and danger to life.
		Flood warning	Flooding is expected from rivers, heavy rain and/or high tides and surges. Immediate action should be taken.

## Data Availability

The code for data loading and BERTopic modelling is available at https://github.com/JGIBristol/ClimateTopicModelling. The code for the rest of the analysis and results visualisation will be available at https://github.com/BrisClimate/.

## References

[R1] ALSPAC (2024). Explore data and samples.

[R2] Ambrose T, Topping A (2024). Wind warnings as 99mph storm isha hits UK.

[R3] Berlemann M, Regner J, Tutt J, Maddison D, Rehdanz K, Welsch H (2020). Handbook on Wellbeing, Happiness and the Environment.

[R4] Bird S, Klein E, Loper E (2009). Natural Language Processing with Python.

[R5] Botzen WJW, Deschenes O, Sanders M (2019). The economic impacts of natural disasters: a review of models and empirical studies. Rev Environ Econ Pol.

[R6] Boyd A, Golding J, Macleod J, Lawlor DA, Fraser A, Henderson J, Molloy L, Ness A, Ring S, Smith GD (2013). Cohort profile: the ‘children of the 90s’-The index offspring of the avon longitudinal study of parents and children. Int J Epidemiol.

[R7] Carbonell J, Goldstein J (1998). The use of MMR, diversity-based reranking for reordering documents and producing summaries.

[R8] Charlton-Perez AJ, Aldridge RW, Grams CM, Lee R (2019). Winter pressures on the UK health system dominated by the Greenland blocking weather regime. Weather Clim Extrem.

[R9] Clair A, Baker E (2022). Cold homes and mental health harm: evidence from the UK household longitudinal study. Soc Sci Med.

[R10] Curtis S, Fair A, Wistow J, Val DV, Oven K (2017). Impact of extreme weather events and climate change for health and social care systems. Environ Health: Global Acc Sci Sour.

[R11] Dell M, Jones BF, Olken BA (2014). What do we learn from the weather? The new climate-economy literature. J Econ Lit.

[R12] Dixon WG, Beukenhorst AL, Yimer BB, Cook L, Gasparrini A, El-Hay T, Hellman B, James B, Vicedo-Cabrera AM, Maclure M, Silva R (2019). How the weather affects the pain of citizen scientists using a smartphone app. npj Digit Med.

[R13] Field A, Miles J, Field Z (2012). Discovering Statistics Using R.

[R14] Fraser A, Macdonald-wallis C, Tilling K, Boyd A, Golding J, Davey smith G, Henderson J, Macleod J, Molloy L, Ness A, Ring S (2013). Cohort profile: the avon longitudinal study of parents and children: ALSPAC mothers cohort. Int J Epidemiol.

[R15] Gasparrini A, Masselot P, Scortichini M, Schneider R, Mistry MN, Sera F, Macintyre HL, Phalkey R, Vicedo-cabrera AM (2022). Small-area assessment of temperature-related mortality risks in England and Wales : a case time series analysis. Lancet Planet Health.

[R16] Godwin JL, Lo YTE, Maude U, Timpson NJ, Northstone K (2025). Extreme heat impacts on daily life and adaptive behaviours captured through lived experience. Environ Res Lett.

[R17] Graham H, White P, Cotton J, McManus S (2019). Flood- and weather-damaged homes and mental health: an analysis using England’s mental health survey. Int J Environ Res Publ Health.

[R18] Grootendorst M (2022). BERTopic: neural topic modeling with a class-based TF-IDF procedure.

[R19] Harris PA, Taylor R, Thielke R, Payne J, Gonzalez N, Conde JG (2009). Research electronic data capture (REDCap)-A metadata-driven methodology and workflow process for providing translational research informatics support. J Biomed Inf.

[R20] Health and Social Care Public Health Agency for Northern Ireland (2024). Keeping warm at home during cold weather.

[R21] Hill R, Griffiths D, Janssen H, Ford K, Carella N, Gascoyne B, Azam S (2024). Cold Homes in Wales: Is the Satisfactory Heating Regime Appropriate for Health and well-being?.

[R22] Hinson S, Bolton P (2024). Fuel Poverty.

[R23] Hollis D, McCarthy M, Kendon M, Legg T, Simpson I (2019). HadUK-Grid—A new UK dataset of gridded climate observations. Geosci Data J.

[R24] Jones CA, Davies SJ, Macdonald N (2012). Examining the social consequences of extreme weather: the outcomes of the 1946/1947 winter in upland Wales, UK. Clim Change.

[R25] Jones L, Mays N (2016). The experience of potentially vulnerable people during cold weather: implications for policy and practice. Public Health.

[R26] Jowkar M, Rijal HB, Montazami A, Brusey J, Temeljotov-Salaj A (2020). The influence of acclimatization, age and gender-related differences on thermal perception in university buildings: case studies in Scotland and England. Build Environ.

[R27] Kendon M (2024a). Storm Henk, 2 January 2024.

[R28] Kendon M (2024b). Storms Isha and Jocelyn, 21 to 24 January 2024.

[R29] Kennedy-Asser AT, Andrews OD, Montgomery J, Jenkins KL, Smith BAH, Lewis E, Birkinshaw SJ, He H, Pywell RF, Brown MJ, Redhead JW (2025). The role of local knowledge in enhancing climate change risk assessments in rural Northern Ireland. Clim Risk Manag.

[R30] Major-Smith D, Heron J, Fraser A, Lawlor DA, Golding J, Northstone K (2022). The avon longitudinal Study of parents and children (ALSPAC): a 2022 update on the enrolled sample of mothers and the associated baseline data. Wellcome Open Res.

[R31] Major-Smith D, Morgan J, Halstead I, Tohidinik HR, Iles-Caven Y, Golding J, Northstone K (2023). Demographic and socioeconomic predictors of religious/spiritual beliefs and behaviours in a prospective cohort study (ALSPAC) in southwest England: results from the parental generation. Wellcome Open Res.

[R32] McInnes L, Healy J (2017). Accelerated hierarchical density clustering.

[R33] McInnes L, Healy J, Melville J (2020). UMAP: uniform manifold approximation and projection for dimension reduction.

[R34] Mitchell DM, Lo YTE, Ball E, Godwin JL, Andrews O, Barciela R, Barrang-Ford L, Di Napoli C, Ebi KL, Fuckar NS, Gasparrini A (2024). Expert judgement reveals current and emerging UK climate-mortality burden. The Lancet Planetary Health.

[R35] Morris S (2024). Storm Henk hits Britain, blocking roads, bridges and rail lines in south.

[R36] Mulchandani R, Armstrong B, Beck CR, Waite TD, Amlôt R, Kovats S, Leonardi G, Rubin GJ, Oliver I (2020). The English national cohort study of flooding & health: psychological morbidity at three years of follow up. BMC Public Health.

[R37] Nguyen HT, Christian H, Le HT, Connelly L, Zubrick SR, Mitrou F (2023). The impact of weather on time allocation to physical activity and sleep of child-parent dyads. Sci Total Environ.

[R38] Northstone K, Ben Shlomo Y, Teyhan A, Hill A, Groom A, Mumme M, Timpson N, Golding J (2023). The avon longitudinal study of parents and children ALSPAC G0 partners: a cohort profile. Wellcome Open Res.

[R39] Northstone K, Lewcock M, Groom A, Boyd A, Macleod J, Timpson N, Wells N (2019). The avon longitudinal study of parents and children (ALSPAC): an update on the enrolled sample of index children in 2019. Wellcome Open Res.

[R40] Public Health Scotland (2024). Protecting the population from the negative health and wellbeing impacts of adverse weather: public health Scotland plan 2024-2027.

[R41] Ratwatte Priyanjali, Wehling H, Kovats S, Landeg O, Weston Dale (2022). Factors associated with older adults’ perception of health risks of hot and cold weather event exposure: a scoping review. Front Public Health.

[R42] Rizmie D, de Preux L, Miraldo M, Atun R (2022). Impact of extreme temperatures on emergency hospital admissions by age and socio-economic deprivation in England. Soc Sci Med.

[R43] Sammut C, Webb GI (2011). Encyclopedia of Machine Learning.

[R44] Seaton-O’Connor A, Wilkes R, Garcia I, Chard R, Sweeney B (2024). Warm Home Prescription® Insights and Impact Report.

[R45] Smith R, Wilson I (2023). Warm homes prescription impact and value for money report.

[R46] Spear M (2025). Can aching joints really predict the weather? Exploring the science behind the stormy debate. The Conversation.

[R47] The Bevan Foundation (2024). A snapshot of poverty in winter 2024.

[R48] The Scottish Government (2017). A New Definition of Fuel Poverty in Scotland.

[R49] Torricelli M, Falkenberg M, Galeazzi A, Zollo F, Quattrociocchi W, Baronchelli A (2023). How does extreme weather impact the climate change discourse? Insights from the Twitter discussion on hurricanes. PLOS Climate.

[R50] Turner GA, Moreira de Sousa A, O’Connell E, Kovats S, Brooks K, Landeg O, Ismail S, Rajamani A, Hajat S (2024). Health Perceptions of Adverse Weather in Older Adults in England: Analysis of 2019/20 Survey Data. European Journal of Public Health.

[R51] UK Health Security Agency (2024a). Adverse Weather and Health Plan.

[R52] UK Health Security Agency (2024b). Adverse Weather and Health Plan: Supporting Evidence 2024.

[R53] UK Health Security Agency (2024c). Cold-health alerts issued by UKHSA and the met office.

[R54] UK Health Security Agency (2024d). Keeping warm and well: staying safe in cold weather.

[R55] UK Met Office (2024a). Seasonal assessment – winter 2024.

[R56] UK Met Office (2024b). Stay safe in a storm.

[R57] UK Met Office Press Office (2024a). Very cold next week.

[R58] UK Met Office Press Office (2024b). A month of contrasts for January’s weather.

[R59] van Hoof J, Schellen L, Soebarto V, Wong JKW, Kazak JK (2017). Ten questions concerning thermal comfort and ageing. Build Environ.

[R60] Wan K, Feng Z, Hajat S, Doherty RM (2022). Temperature-related mortality and associated vulnerabilities: evidence from Scotland using extended time-series datasets. Environ Health: Global Acc Sci Sour.

[R61] Zivin JG, Neidell M (2014). Temperature and the allocation of time: implications for climate change. J Labor Econ.

