# Supplementary material for: Sociodemographic vulnerability to cold and stormy weather in relation to health and life
Source: Weather Clim Extrem. Author manuscript; Available in PMC 2026 Sep 7. (PMC7619283; doi:10.1016/j.wace.2025.100840)
Supplement: Supplementary file 1 [file EMS215452-supplement-Appendix_A__Supplementary_data.docx]

**Sociodemographic vulnerability to cold and stormy weather impacts on health and life**

Y. T. Eunice Lo, Joanne Godwin, Ulrika Maude, Huw Day, Nicolas Timpson, Kate Northstone

Supplementary information

1. **Survey questions**

**Introduction**

We are interested in understanding how you might be affected by extreme weather events. For some people, a particularly wet day might bring a minor inconvenience; for others, it can be hugely disruptive. In January, we experienced a period of particularly cold weather, as well as Storms Henk, Isha and Jocelyn, and would like to ask you some questions about how you coped during these.

Please remember that this survey is completely anonymous; we will not be able to link your responses to any other data you have given to us as part of Children of the 90s. If you provide any personal information in any of the free text responses, this will be anonymised before the survey results are shared with any researchers.

**About the extreme cold weather the UK in January 2024:**

We would like you to think back to the days between 14^th^ and 20^th^ January 2024, during which the UK experienced close to freezing daytime temperatures and below freezing nights over several days. If you were not in England during this period, the survey will not apply to you. We understand this may be difficult to remember, but all your responses are valuable.

1. How were different aspects of your life affected by this period of cold weather, compared to normal?

|  | A lot worse | Slightly worse | No change | Slightly better | A lot better | N/A |
| --- | --- | --- | --- | --- | --- | --- |
| Mood |  |  |  |  |  |  |
| Productivity at home |  |  |  |  |  |  |
| Productivity at work |  |  |  |  |  |  |
| Sleep quality |  |  |  |  |  |  |
| Appetite |  |  |  |  |  |  |
| Physical health |  |  |  |  |  |  |
| Physical activity / exercise |  |  |  |  |  |  |
| Access to GP, hospital or emergency care |  |  |  |  |  |  |

2. Were you aware of the cold weather warnings?	Yes | No

3. Did you make any changes to your usual daily activities in order to deal with the cold temperatures? For example, work from home, change your usual mode of transport, cancel personal or professional appointments, buy a heater.

a. I made changes in response to the cold warning (i.e. in advance of cold weather)	Yes | No

If yes, please tell us, what changes you made	Free text

b. I made changes in response to the cold weather itself	Yes | No

If yes, please tell us, what changes you made	Free text

4. Is there anything else you would like to tell us about how you were affected by this period of particularly cold weather?

Free Text

**About Storms Henk, Isha and Jocelyn in the UK in January 2024:**

In January 2024 the UK also experienced three named storms. If you were not in the UK during this period the survey will not apply to you. We understand this may be difficult to remember, but all your response are valuable.

5. How were different aspects of your life affected by this period of heavy rain and strong wind, compared to normal?

|  | A lot worse | Slightly worse | No change | Slightly better | A lot better | N/A |
| --- | --- | --- | --- | --- | --- | --- |
| Mood |  |  |  |  |  |  |
| Productivity at home |  |  |  |  |  |  |
| Productivity at work |  |  |  |  |  |  |
| Sleep quality |  |  |  |  |  |  |
| Appetite |  |  |  |  |  |  |
| Physical health |  |  |  |  |  |  |
| Physical activity / exercise |  |  |  |  |  |  |
| Access to GP, hospital or emergency care |  |  |  |  |  |  |

6. Were you aware of the storm or flood warnings? Yes | No

7. Did you make any changes to your usual daily activities in order to deal with the storms? For example, work from home, change your usual mode of transport, cancel personal or professional appointments, buy a dehumidifier.

a. I made changes in response to the storm warning (i.e. in advance of the storm). Yes | No

If yes, please tell us, what changes you made	Free text

b. I made changes in response to the cold weather itself	Yes | No

If yes, please tell us, what changes you made	Free text

8. Is there anything else you would like to tell us about how you were affected by this period of particularly stormy weather?

Free Text

**About you**

9. What is your gender?

Male | Female | Non-binary | Prefer not to say | Other (text box)

10. What is your age?

25-34 | 35-44 | 45-54 | 55-64 | 65-74 | 75-84 | 85+ | Prefer not to say

11. Do you have a disability?

Yes | No | Prefer not to say

12. Do you have a long-term health condition? i.e. diabetes, arthritis

Yes | No | Prefer not to say

13. What type of housing do you live in?

Flat | Detached or semi-detached house | Terraced house | Bungalow | Prefer not to say |

Other (text box)

14. How would you describe the area where you live?

City or town centre | City or town suburbs| Rural/Countryside/Coast | Prefer not to say |

Other (text box)

15. Which region of the UK do you live in?

Cymru Wales | East Midlands | East of England | London | North East & Cumbria | North West | Northern Ireland | Scotland | South East | South West | West Midlands | Yorkshire & the Humber

16. What is your job status?

Employed | Self-employed | Unemployed or not working | Student | Retired | Prefer not to say | Other (text box)

17. I understand that by submitting the information I have provided above, my data cannot be identified and attributed to me and therefore cannot be deleted. It will not be linked to the rest of Children of the 90s data.

Tick box to indicate

2. **Main analysis supplementary information**

**Table S1.** Minimum adequate regression models in the cold lived experience analysis.

| **Aspect of life** | **Minimum adequate model** |
| --- | --- |
| Physical health | RESPONSEX ~ GNDR + AGE4 + DISAB + LTHC |
| Physical activity | RESPONSEX ~ AGE4 + AREA4 + LTHC |
| Sleep quality | RESPONSEX ~ (GNDR:AGE4) + AGE4 + JOB4 + DISAB + LTHC |
| Appetite | RESPONSEX ~ GNDR + AGE4 + JOB4 + DISAB + LTHC |
| Mood | RESPONSEX ~ GNDR + AGE4 + LTHC |
| Productivity at home | RESPONSEX ~ GNDR + AGE4 + JOB4 + LTHC |
| Productivity at work | RESPONSEX ~ AGE4 + JOB4 + AREA4 + LTHC |
| Access to healthcare | RESPONSEX ~ GNDR + DISAB + LTHC |

**Table S2.** Odds ratios (ORs), 95% confidence intervals (CIs) and associated *p* values of reporting worse lived experience (versus no change), during cold weather in sociodemographic groups compared to corresponding reference (ref) groups. Only variables that are included in minimum adequate models in Table S1 are listed here.

| **Group** | **Ref group** | **Aspect of life** | **OR** | **95% CI** | **P value** |
| --- | --- | --- | --- | --- | --- |
| Female | Male | Physical health | 1.5 | 1.2 – 1.8 | 0.0003 |
|  |  | Appetite | 2.8 | 2 – 3.9 | 1 x 10^-9^ |
|  |  | Mood | 1.4 | 1.2 – 1.6 | 0.0003 |
|  |  | Productivity at home | 1.5 | 1.3 – 1.8 | 5 x 10^-6^ |
|  |  | Access to healthcare | 1.5 | 1.1 – 2.2 | 0.02 |
| Age ≤ 34 | Age ≥ 65 | Physical health | 1.7 | 1.3 – 2.3 | 2 x 10^-5^ |
|  |  | Physical activity | 1.3 | 1 – 1.6 | 0.02 |
|  |  | Sleep quality | 2.1 | 1.3 – 3.4 | 0.003 |
|  |  | Appetite | 2.1 | 1.3 – 3.4 | 0.001 |
|  |  | Mood | 2.2 | 1.7 – 2.7 | 8 x 10^-12^ |
|  |  | Productivity at home | 1.6 | 1.2 – 2.2 | 0.001 |
|  |  | Productivity at work | 2.1 | 1.4 – 3.3 | 0.0004 |
| Age 35-64 | Age ≥ 65 | Physical health | 1.3 | 1 – 1.6 | 0.05 |
|  |  | Physical activity | 1 | 0.8 – 1.2 | 0.7 |
|  |  | Sleep quality | 0.7 | 0.4 – 1.2 | 0.2 |
|  |  | Appetite | 1.2 | 0.8 – 1.8 | 0.4 |
|  |  | Mood | 0.9 | 0.8 – 1.2 | 0.6 |
|  |  | Productivity at home | 0.8 | 0.5 – 1.3 | 0.9 |
|  |  | Productivity at work | 1.1 | 0.8 – 1.7 | 0.6 |
| Female ≤ 34 | Male ≥ 65 | Sleep quality | 1.2 | 0.9 – 1.7 | 0.2 |
| Female 35-64 | Male ≥ 65 | Sleep quality | 2.5 | 1.7 – 3.6 | 7 x 10^-6^ |
| Female >= 65 | Male ≥ 65 | Sleep quality | 1.4 | 0.9 – 2.1 | 0.1 |
| Long-term health condition | No long-term health condition | Physical health | 2.4 | 2 – 2.9 | 0.0 |
|  |  | Physical activity | 1.6 | 1.3 – 1.9 | 2 x 10^-7^ |
|  |  | Sleep quality | 1.5 | 1.2 – 1.8 | 0.0005 |
|  |  | Appetite | 1.6 | 1.2 – 2.1 | 0.001 |
|  |  | Mood | 1.3 | 1.1 – 1.6 | 0.003 |
|  |  | Productivity at home | 1.5 | 1.3 – 1.8 | 6 x 10^-6^ |
|  |  | Productivity at work | 1.4 | 1.1 – 1.8 | 0.002 |
|  |  | Access to healthcare | 2 | 1.4 – 2.7 | 4 x 10^-5^ |
| Disability | No disability | Physical health | 2 | 2 – 2.9 | 1.6 x 10^-5^ |
|  |  | Sleep quality | 1.9 | 1.3 – 2.7 | 0.0003 |
|  |  | Appetite | 2.4 | 1.6 – 3.6 | 2 x 10^-5^ |
|  |  | Access to healthcare | 2.7 | 1.8 – 4.1 | 4 x 10^-6^ |
| Unemployed/not working | Retired | Sleep quality | 1.8 | 1.1 - 3 | 0.03 |
|  |  | Appetite | 1.4 | 0.7 – 2.7 | 0.4 |
|  |  | Productivity at home | 2 | 1.3 – 3.3 | 0.03 |
|  |  | Productivity at work | 2.8 | 1.2 – 6.6 | 0.02 |
| Employed/self employed | Retired | Sleep quality | 1.2 | 0.9 – 1.6 | 0.2 |
|  |  | Appetite | 1.7 | 1.1 – 2.5 | 0.01 |
|  |  | Productivity at home | 1.1 | 0.9 – 1.4 | 0.5 |
|  |  | Productivity at work | 2.9 | 1.8 – 4.8 | 0.0 |
| Urban area | Rural area | Physical activity | 1.1 | 0.9 – 1.5 | 0.4 |
|  |  | Productivity at work | 1.2 | 0.8 – 1.6 | 0.4 |
| Suburban area | Rural area | Physical activity | 1 | 0.9 – 1.2 | 0.8 |
|  |  | Productivity at work | 0.8 | 0.6 - 1 | 0.03 |

**Table S3.** Same as Table S1 but for the storms lived experience analysis.

| **Storms** | **Minimum adequate model** |
| --- | --- |
| Physical health | RESPONSEX ~ GNDR + AGE4 + DISAB + LTHC |
| Physical activity | RESPONSEX ~ (GNDR:AGE4) + GNDR + AGE4 + LTHC |
| Sleep quality | RESPONSEX ~ GNDR + AGE4 + DISAB + LTHC |
| Appetite | RESPONSEX ~ GNDR + AGE4 + DISAB + LTHC |
| Mood | RESPONSEX ~ GNDR + AGE4 + LTHC |
| Productivity at home | RESPONSEX~ GNDR + AGE4 + JOB4 + LTHC |
| Productivity at work | RESPONSEX ~ AGE4 + JOB4 |
| Access to healthcare | RESPONSEX ~ GNDR + DISAB + LTHC |

**Table S4.** Odds ratios (ORs), 95% confidence intervals (CIs) and associated *p* values of reporting worse lived experience (versus no change), during stormy weather in sociodemographic groups compared to corresponding reference (ref) groups. Only variables that are included in minimum adequate models in Table S3 are listed here.

| **Group** | **Ref group** | **Aspect of life** | **OR** | **95% CI** | **P value** |
| --- | --- | --- | --- | --- | --- |
| Female | Male | Physical health | 1.5 | 1.2 – 1.9 | 0.0002 |
|  |  | Physical activity | 1.7 | 1.3 – 2.4 | 0.0009 |
|  |  | Sleep quality | 1.8 | 1.5 – 2.2 | 7 x 10^-10^ |
|  |  | Appetite | 2.8 | 1.9 – 3.9 | 3 x 10^-8^ |
|  |  | Mood | 1.9 | 1.6 – 2.3 | 7 x 10^-14^ |
|  |  | Productivity at home | 1.6 | 1.4 - 2 | 6 x 10^-7^ |
|  |  | Access to healthcare | 1.8 | 1.2 – 2.6 | 0.003 |
| Age ≤ 34 | Age ≥ 65 | Physical health | 1.6 | 1.2 – 2.2 | 0.0006 |
|  |  | Physical activity | 0.9 | 0.6 – 1.3 | 0.5 |
|  |  | Sleep quality | 1.5 | 1.2 – 1.9 | 0.0006 |
|  |  | Appetite | 2.4 | 1.6 – 3.5 | 2 x 10^-5^ |
|  |  | Mood | 1.8 | 1.5 – 2.3 | 1 x 10^-7^ |
|  |  | Productivity at home | 1.6 | 1.2 – 2.2 | 0.002 |
|  |  | Productivity at work | 1.7 | 1.1 – 2.6 | 0.01 |
| Age 35-64 | Age ≥ 65 | Physical health | 1.3 | 1 – 1.7 | 0.07 |
|  |  | Physical activity | 1.3 | 0.9 – 1.8 | 0.2 |
|  |  | Sleep quality | 1.1 | 0.9 – 1.4 | 0.2 |
|  |  | Appetite | 1.2 | 0.8 – 1.8 | 0.3 |
|  |  | Mood | 1 | 0.8 – 1.2 | 1 |
|  |  | Productivity at home | 0.9 | 0.7 – 1.1 | 0.3 |
|  |  | Productivity at work | 1 | 0.6 – 1.5 | 0.9 |
| Female ≤ 34 | Male ≥ 65 | Physical activity | 1 | 0.6 – 1.6 | 1 |
| Female 35-64 | Male ≥ 65 | Physical activity | 0.6 | 0.4 – 0.9 | 0.01 |
| Long-term health condition | No long-term health condition | Physical health | 2.3 | 1.8 – 2.8 | 6 x 10^-14^ |
|  |  | Physical activity | 1.4 | 1.2 – 1.7 | 6 x 10^-5^ |
|  |  | Sleep quality | 1.3 | 1.1 – 1.6 | 0.003 |
|  |  | Appetite | 1.7 | 1.3 – 2.3 | 0.0003 |
|  |  | Mood | 1.3 | 1.1 – 1.5 | 0.01 |
|  |  | Productivity at home | 1.6 | 1.3 – 2 | 5 x 10^-7^ |
|  |  | Access to healthcare | 2.1 | 1.5 – 2.9 | 3 x 10^-5^ |
| Disability | No disability | Physical health | 2.5 | 1.8 – 3.4 | 9 x 10-8 |
|  |  | Sleep quality | 1.6 | 1.2 – 2.3 | 0.003 |
|  |  | Appetite | 2.3 | 1.7 – 3.9 | 4 x 10^-6^ |
|  |  | Access to healthcare | 2.5 | 1.6 – 3.9 | 5 x 10^-5^ |
| Unemployed/not working | Retired | Productivity at home | 1.9 | 1.2 – 3 | 0.01 |
|  |  | Productivity at work | 2.9 | 1.2 – 6.8 | 0.02 |
| Employed/self employed | Retired | Productivity at home | 1 | 0.8 – 1.3 | 0.7 |
|  |  | Productivity at work | 2.8 | 1.7 – 4.6 | 5 x 10^-5^ |

3. **Sensitivity analysis supplementary information**

**Figure S1.** Same as Figure 2 in the main manuscript, but these results are generated from the subset of respondents who are based in South West England (n = 2272 respondents).

**Figure S2.** Same as Figure 3 in the main manuscript, but these results are generated from the subset of respondents who are based in South West England (n = 2272 respondents).

**Figure S3.** Same as Figure 4 in the main manuscript, but these results are generated from the subset of respondents who are based in South West England (n = 2272 respondents).
